# Aural Syringes

**Published:** 1880-07-20

**Authors:** J. S. Prout


### AURAL SYRINGES.

BY J. S. PROUT, M. D.

As announced by the card, I shall confine my remarks to ear syringes, saying very little on the general subject of ear syringing, and shall address them to those who, as general practitioners, have not time or opportunity to make themselves expert in matters of aural practice. I hope that what I say may also interest our friends, the druggists, one of whom recently told me that "ear syringes" are sometimes bought by men who propose to use them on an entirely different part of the body!

As the subject is an important one, our patients are entitled to explicit instructions from us in respect to indications, instruments, etc. I therefore lay it down as a rule, from which there is to be no departure, that no ear should be actively syringed unless there is something in it that it is important to remove. It therefore follows, as a matter of course, that if the physician cannot examine the ear and determine that there is something in it that needs to be removed, he is not competent to advise for the case; and also that if, in despite of this inability, he prescribes syringing, he does so blindly, at hap-hazard, ignorantly, and is guilty of malpractice (bad practice), even though no appreciable injury is sustained by the patient.

What then are the things that we are to remove from the ear? They are foreign bodies, however they may have gotton in, wax and pus, which last may be either dried and caked in the auditory canal, or in process of constant formation and discharge.

Nearly all the objects that enter the ear from without may be removed by means of the syringe, and no one who has not given careful attention to the treatment of aural diseases should ever resort to any other method of removal.

When one reads and hears of the horrid work that has been and is still occasionally done by legally qualified practitioners in the search after foreign bodies that perhaps have no existence—drum-membranes destroyed, ossicles pulled out, etc., etc.—one is tempted to wish that some such unscrupulous imcompetent may be sued for malpractice and mulched in heavy damages—*pour eneaurager les autres!*

For the purpose of classification I shall divide syringes into 1, piston-syringes; 2, bulb-syringes in which the injecting nozzle is directly attached to the bulb; and 3, bulb syringes in which the nozzle is connected with the bulb by a flexible rubber tube of several inches in length, the water being drawn into the bulb through a similar flexible tube, as in Davidson's, or through a short metallic tube, as in the Derby or the American.

In the drug stores small cheap glass or pewter piston-syringes (made originally for the penis, perhaps) are sold for the ear, for which they are dangerous and almost worthless. I was told by a druggist the other day that a small glass syringe of this sort broke recently in a lady's ear while in use. The result was a cut on the cheek—strangely enough and very fortunately, the ear itself was not injured. Small hard-rubber piston-syringes of various styles are also sold for the ear, one of which, called the "ear syringe, is, in careful hands, a good instrument. I have used one of this sort, holding about ½ ounce, for years, and find it sufficient for all ordinary purposes. When the piston does not work freely there is apt to be a jerky movement, and the delicate skin lining the auditory canal is apt to be injured. It is also true that no one can use such an instrument satisfactorily and safely on himself. I therefore make the broad general statement that for home and self use, on the ear, a piston-syringe should not be used. It is both dangerous and unhandy.

The different bulb-syringes of the first variety (second class) are better than tnose of which I have just spoken (first class), but they are less safe and less easily used than those of class 3, of which Davidson's may be ranked as first and best, the type of the good syringe for the ear for home or self use. The power is applied by the hand to the bulb, and the force of the stream can be regulated with great nicety. The intervention of the flexible tube between the bulb and the nozzle prevents any possibility of damage through the force exerted by the hand, as may easily happen in using the piston-syringes. The nozzle may be held by the syringer or by the patient, with its point in the beginning of the external meatus. Any sudden movement of the head will be away from danger. This syringe, or one of this class, is to be found in almost every well-regulated household.

Another advantage of this class (3) is that when the syringe is in action, if the source of water-supply be raised it will act as a douche and give a steady stream of water to the ear, the force varying as the difference of level between nozzle and water-surface is great or small. Thus the Davidson syringe, besides its ordinary uses, is the best attainable domestic ear-syringe, and is as good a douche for the ear as we need, while the others of the third class are nearly as good.

The last addition to the family is Hall's Patent Health Syringe, which may be put into our third class. A glass jar for holding the water has attached to its top a rubber bulb by means of which the air is condensed in the space above the water, which, through a tube that goes to the bottom of the jar and terminates externally in a flexible tube, is thus forced out in a steady stream at a moderate pressure.

What fluid is to be used in the syringe? Warm water of about the temperature of the blood (98½° Fahrenheit) is sufficient for all ordinary purposes. (Nothing cold should ever be put in the ear.) When there is a purulent discharge, a solution of borax (one or two teaspoonfuls to the pint) may be used, as it is both cleansing and healing. This or a solution of carbonate or bicarbonate of soda is sometimes used to soften wax, as the alkali somewhat increases the solvent power of the water; but this is seldom needed. Castile soap may be added to the water; but, as a rule, it is better to avoid fluids containing organic material. (For the same reason it is unwise in earache to pour in oils, molasses or such substances, as they make the ear dirty, and so favor the developement of disease. Warm water or this, with three times its bulk of laudanum, will usually do more good and no harm.)

It is essential that only clean water be taken into the syringe. This is easily managed by using two vessels, one a cup or finger-bowl to catch the water that flows from the ear, while in the other the clean water is held. By this precaution the syringe is always clean, and cannot carry infection from one patient to another.

Syringing the ear may sometimes produce disagreeable effects, such as cough, giddiness, or even fainting. Among other causes these may result from the use of water of too high temperature. Dr. Cornwell mentions a case in which faintness came on whenever the water was "a little more than milk-warm." (Cincinnati Lancet and Clinic, Nov. 8, 1879, p. 360.)

For ordinary syringing the temperature may be about 98° Fahr.; for the douche, for the relief of acute inflammation, it may be somewhat warmer, but in any case the feelings of the patient are the only safe guide.

Roosa (Diseases of the Ear, 4th Edition, 1878, p. 127) says: "I think the small hard rubber syringe is the best, though a Davidson's syringe does very well." Turnbull (Dis. of the Ear, Phila., 1872, p. 86) advises Davidson's syringe, and says if this is not at hand a piston syringe (described and figured) may be substituted. I am sorry to say that he violates what I hold to be a cardinal rule in ear-syringing when he says (p. 89): "Wilde's basin is of great service in these cases. Its concave part fits accurately the curve beneath the lobe of the ear, and the perforated septum *strains the clean water from the dirty.*" (Italics mine.) This is disagreeably suggestive of dirty instruments, and of the communication of disease from one patient to another. Von Troeltsch says: "There can be but one object in syringing the ear; that is, the removal of something from it. You will be still more surprised when in your practice you find that almost every aural patient, who has not come to you at first, has been ordered to syringe the ears. The patients who tell you this will often very earnestly and truly tell you that nothing was removed." (Trans. by Roosa, 2d Am. Edition, N. Y., 1869, p. 93.) Burnett (Diseases of the Ear, Phila., 1877) only mentions the piston syringe, and repeats the same advice in his "Hearing; and How to Keep It" (American Health Primer, No. 1, Phila., 1879) an excellent little book, of interest to us all, and one that I wish I could induce every member of this Society to read. It would then be well for them and better for their patients. I also wish that it could receive the wide general reading that it deserves, and for which it was intended. Those with ear trouble would then better appreciate the quality of the advice they receive. Light is good in dark places. The influence for good on the profession, of correct ideas among the laity, cannot be overestimated.

In conclusion, let me say that I always protect the reputation of a professional brother if possible. Let me cite a case to show why this cannot always be done: A very intelligent patient came to me for treatment, saying that his physician had assured him that there was no wax in his ears. I found each ear nearly full of wax, which I was obliged to syringe out. I did not comment on this unfortunate contradiction, nor did he, but it is not safe to infer that he did not notice it, and did not draw his own conclusions.

The instinct of self-preservation prompts patients to respect a physician who candidly says he does not understand some disease of theirs that is entirely out of his line, and he maintains his own self-respect by doing so. But how does the case stand when the patient discovers that the physician professes to understand matters concerning which he is perhaps entirely uninformed? Should not each man know and honestly recognize his own limitations?—*Soc. Co. Kings.*

